# Insulin-like growth factor-1 receptor participates in the anti-MDS action involving the MICA/B-NKG2D axis

**DOI:** 10.3389/fonc.2026.1960550

**Published:** 2026-10-29

**Authors:** Xiaohan Liu, Lei Huang, Yixuan Guo, Juan Mu, Xueke Zhu, Zhaoyun Liu, Rong Fu

**Affiliations:** 1 Department of Hematology, Tianjin Medical University General Hospital, Tianjin, China; 2 Tianjin Key Laboratory of Bone Marrow Failure and Malignant Hemopoietic Clone Control, Tianjin, China; 3 Tianjin Institute of Hematology, Tianjin, China

**Keywords:** haematology, myelodysplastic syndrome, insulin-like growth factor-1 receptor, MICA/B, NK cells

## Abstract

**Purpose:**

Myelodysplastic syndromes (MDS) represent a clonal malignancy of the hematopoietic system with a high risk of progression to acute myeloid leukemia (AML). Patients with MDS commonly exhibit natural killer (NK) cell dysfunction, impairing their ability to effectively eliminate malignant cells and posing a serious health threat. The key to treating MDS and preventing its progression to AML lies in restoring and activating NK cell killing functions while eliminating malignant cells, thereby establishing immune interactions between MDS cells and NK cells.

**Method:**

We screened the bone marrow of three patients with primary MDS and three patients with MDS converted to AML for relevant differential genes by Nanostring technology and screened for IGF-1R. We explored the potential and mechanisms of targeting IGF-1R in the treatment of MDS by flow cytometry, immunofluorescence, Western blot, and cell co-culture techniques.

**Results:**

Nanostring sequencing results showed differential expression of IGF-1R in bone marrow cells from MDS patients and trans-AML patients. Differential expression was found to be concentrated on CD34+ cells by flow cytometry. Inhibition of IGF-1R induced DNA damage in MDS cell lines SKM-1 and KG-1, upregulated MICA/B on the surface of MDS cells, and enhanced NK cell-mediated killing of MDS cells, consistent with the involvement of the MICA/B–NKG2D axis.

**Conclusion:**

IGF-1R is involved in the genesis and development of MDS and may modulate the interaction between MDS cells and NK cells, thus serving as a potential target for MDS therapy.

## Introduction

Myelodysplastic syndrome (MDS) is a malignant hematopoietic stem/progenitor cell clonal disorder (1–3). The pathogenesis of MDS involves a variety of factors, including molecular abnormalities, cytogenetic and immunological disorders (4–7). In addition to molecular-genetic abnormalities of hematopoietic stem cells, the depletion of immune cells is crucial in the pathogenesis of MDS. An imbalance in the patient’s immune surveillance system and impaired clearance of aberrant clones leads to immune escape and immune tolerance of malignant clonal cells, which is one of the important mechanisms by which MDS patients progress to acute myeloid leukemia (AML) (8–10). Therefore, restoration of the tumor-killing function of patients’ immune cells, especially NK cells, and enhancement of the interaction between NK cells and MDS cells are crucial for treating MDS and preventing its progression to AML (11, 12). The killing of MDS cells and the activation of NK cells have been partially investigated, but no target has been identified that can act on both types of cells and promote their interaction.

The insulin-like growth factor (IGF) signaling pathway is a complex network consisting of 2 ligands (IGF-1 and IGF-2), 2 receptors (IGF-1R and IGF-2R), and 6 IGF-binding proteins (IGFBP1-6) (13–15). IGF-1R is a key participant in a variety of physiological processes such as cell growth, proliferation, differentiation, and apoptosis, and has recently been identified to play a key role in the formation and progression of cancer, including cancer formation, progression and metastasis (16, 17). In addition, clinical studies have shown that IGF-1R has potent anti-apoptotic and transforming activities, and that increased IGF-1R activity is associated with tumor metastasis, treatment resistance, poor prognosis and shorter survival (18–20). Therefore, IGF-1R has become a potential and promising diagnostic and therapeutic biomarker in cancer. However, its role in malignant hematologic diseases has not been investigated. Some previous studies have demonstrated that IGF-1 also has a certain positive regulatory effect on the function of immune cells (21), but the study on the role of IGF-1R in regulating NK cells is still in the exploratory stage, and its regulation of NK cell function is inconclusive. Exploring what kind of role IGF-1R plays in MDS and what kind of regulatory role it has between the interaction of malignant cells and NK cells can provide new ideas and targets for treatment from the perspective of restoring the dysregulation of bone marrow immune microenvironment in MDS patients.

## Materials and methods

### Patients

This study included 20 healthy donors, 20 newly diagnosed myelodysplastic syndrome (MDS) patients and 20 newly diagnosed acute myeloid leukemia (AML) patients from Tianjin Medical University General Hospital, diagnosed and treated between 2023 and 2024. Newly diagnosed MDS patients are classified according to 2022 World Health Organization (WHO) and International Consensus (ICC) classifications (22, 23), and newly diagnosed AML patients are classified according to the FAB classification system. Patient information is summarized in **Supplementary Data Table S1**. The study was approved by the Institutional Review Board (IRB) of Tianjin Medical University (IRB2025-KY-618).

### Nanostring sequencing

Three newly diagnosed patients with myelodysplastic syndrome (MDS) and three patients with secondary acute myeloid leukemia (MDS-AML) were included, and bone marrow samples were collected. Total RNA was extracted using the Trizol, and genomic DNA contamination was removed by DNase I digestion, and samples were aliquoted and stored at -80 °C. The nCounter^®^ PanCancer Immune Profiling panel was used, which includes 770 cancer-related genes. For each sample, 100 ng of total RNA was hybridized with CodeSet probes at 65 °C for 16 hours. The hybridization complex was automatically purified using the nCounter Prep Station and fixed on a cartridge; the nCounter Digital Analyzer scanned the fluorescent barcodes to obtain single-molecule counting raw data. Samples with FOV counting <50% or positive control linearity R² <0.95 were excluded; the stability of housekeeping gene expression was validated using the geNorm algorithm before normalization and differential analysis.

### Reagents

Materials for cell culture were sourced from Nest (Wuxi, China). OSI-906 and IGF-1 were all ordered from MedChemExpress (Monmouth Junction, NJ, USA) and stored as stock solutions at -80 °C. OSI-906 (linsitinib), a selective dual inhibitor of IGF-1R and the insulin receptor (cell-free IC50: 35 nM for IGF-1R and 75 nM for IR), was used at 1 μM, with treatment durations of 48 h for functional assays and 72 h for co-culture experiments. IGF-1 was used at 100 ng/mL, and cells were treated with IGF-1 for 48 h, either alone or in combination with OSI-906.

### Cell culture

SKM-1 cells were cultured in RPMI 1640 supplemented with 1% penicillin/streptomycin (Solarbio, Beijing, China) and 15% fetal bovine serum (Gibco Life Technologies, Carlsbad, CA, USA). KG-1 cells were cultured in RPMI 1640 supplemented with 1% penicillin/streptomycin (Solarbio, Beijing, China) and 20% fetal bovine serum (Gibco Life Technologies, Carlsbad, CA, USA). Cells were cultured in T25 flasks with a coating density of 1 × 10^6 cells/cm2 in humidified air with 5% CO2 at 37°C. All experiments were performed using seed cells with a logarithmic growth phase of 2 × 10^5 cells/mL.

### EdU assay

The EdU cell proliferation assay kit (RiboBio, China) was employed to measure cell proliferation. Cells were incubated with 200 μL of 5-ethynyl-20-deoxyuridine at 37°C for 2 hours. After fixation and permeabilization, the cells were incubated with the Apollo reagent for 30 minutes, and fluorescence intensity was assessed using flow cytometry.

### Tunel assay

The Tunel assay was performed using the Beyotime Biotechnology Tunel Kit (cat. No. C1088). Cells were treated for 48 hours then fixed with 4% of paraformaldehyde for 30 minutes, permeabilized with 0.5% Triton X-100 for 10 minutes, and then incubated with the Tunel assay solution for 60 minutes at 37 °C in the dark. Afterward, cells were stained with DAPI (Solarbio) for 5 minutes and observed under Zeiss Axio Observer 5 inverted microscope (Carl Zeiss Microscopy GmbH). Images were digitally merged to analyze co-localization.

### Comet assay

A comet assay kit (Beyotime Biotechnology; cat. No. C2041M) was used for this analysis. After treated with OSI-906 for 48 h, pre-warmed 1% normal melting point agarose gels were spread on pre-warmed comet electrophoresis slides according to the manufacturer’s protocols, covered with a coverslip, and left at 4 °C for 10 min to allow them to solidify. 10 μL of cells and 75 μL of 0.7% low melting point agarose in a 37 °C water bath were mixed well and added dropwise to the first layer of gel, covered with a coverslip and left at 4 °C for 10 minutes to solidify. The third layer of gel was the same as the first layer. The coverslip was removed and immersed in pre-cooled lysis buffer and lysed at 4 °C for 1–2 h. The slides were removed and rinsed in PBS for 3 min. The slides were immersed in electrophoresis buffer and left at room temperature for 20–60 min. Electrophoresis was performed at low voltage for 20 min. Neutral buffer was added to the slides at 4 °C. About 20 μL of Propidium Iodide Solution was added dropwise to the slides and stained for 10–20 min away from light. The slides were then washed 3 times with Ultrapure Water, covered with a coverslip and then visualized under a fluorescence microscope. Images were analyzed using ImageJ software, and the mean tail length (μm) was quantified for at least 50 cells per group from three independent experiments.

### Flow cytometry analysis

#### NK cells functional analysis

NK-92 cells and SKM-1 cells were co-cultured at an effector-to-target (E:T) ratio of 1:4 for 72 h in the presence or absence of OSI-906 (1 μM). OSI-906 was added to the co-culture system and therefore acted on both cell populations. Cells were washed with cold PBS and stained with the relevant antibodies for 15 minutes at room temperature in the dark. NK-92 cells were identified as CD3−CD56+ and SKM-1 cells as CD117+; NK cell functional markers (NKG2D, CD107a, and perforin) were assessed within the CD3−CD56+ gate, and apoptosis of SKM-1 cells was assessed by Annexin V/PI staining within the CD117+ gate. Intracellular cytokine staining was performed using the BD IntraSurTM Kit. Antibodies used for flow cytometry analysis are listed in **Supplementary Table S2**.

#### Apoptosis of cells

Apoptosis of cells was assessed by flow cytometry using the BD PharmingeTM FITC Annexin V Apoptosis Detection Kit I, following the manufacturer’s instructions.

### Western blot

Cell lysates were prepared using RIPA buffer (Solarbio, China) containing protease and phosphatase inhibitors (Solarbio, China). Protein concentrations were determined by the Bradford assay. Protein samples were mixed with 5X loading buffer (Beyotime, China), boiled at 100 °C for 10 minutes, and then separated by SDS-PAGE. Proteins were transferred to polyvinylidene fluoride (PVDF) membranes and blocked with 5% skimmed milk. Membranes were incubated with primary and secondary antibodies, and protein bands were visualized using the BLT GelView 6000Plus Imaging System. Quantification and analysis of the protein bands were performed using ImageJ software. Primary and secondary antibodies are listed in **Supplementary Table S3**.

### Statistical analysis

All *in vitro* experiments were performed with n = 3 independent biological replicates. Data are presented as mean ± SD. Normality of data distribution was assessed using the Shapiro–Wilk test. Comparisons among three groups were performed using one-way ANOVA followed by Tukey’s *post hoc* test for multiple-comparison correction. A p value < 0.05 was considered statistically significant. Statistical analyses were performed using GraphPad Prism software.

## Results

### IGF-1R is differentially expressed in bone marrow cells from MDS patients and MDS-AML patients, with the main differences observed in CD34+ cells

We included 3 patients with MDS and 3 patients with MDS-AML, and performed Nanostring sequencing on all bone marrow cells from these patients. The sequencing results showed that IGF-1R expression was significantly upregulated in bone marrow cells from MDS-AML patients (**Figures 1A, B**), suggesting a potential role for IGF-1R in the development of MDS and its progression to AML. Because NanoString sequencing was performed on bulk bone marrow cells and cannot resolve which cell populations account for the differential signal, we next measured IGF-1R surface expression on individually gated bone marrow subsets by flow cytometry, an approach independent of the cellular composition of the bulk marrow samples. To further identify the specific cells with differentially expressed IGF-1R, we included 20 healthy controls (HC), 20 MDS patients, and 20 AML patients (detailed clinical information of the patients is provided in **Supplementary Data Table S1**). We measured the expression levels of IGF-1R on the surface of CD34+ cells, NK cells, Treg cells, CD4+ T cells, CD8+ T cells, and MDSCs (**Supplementary Figure S1**). Flow cytometry results showed that differential expression of IGF-1R was primarily observed on the surfaces of CD34+ cells. Compared to HC, MDS patients exhibited increased IGF-1R expression on the surfaces of CD34+ cells. AML patients also showed increased IGF-1R expression on the surfaces of CD34+ cells compared to MDS patients (**Figures 1C, D**). Therefore, the differential expression of IGF-1R on the surfaces of CD34+ cells is likely involved in the onset and progression of MDS.

**Figure 1:**
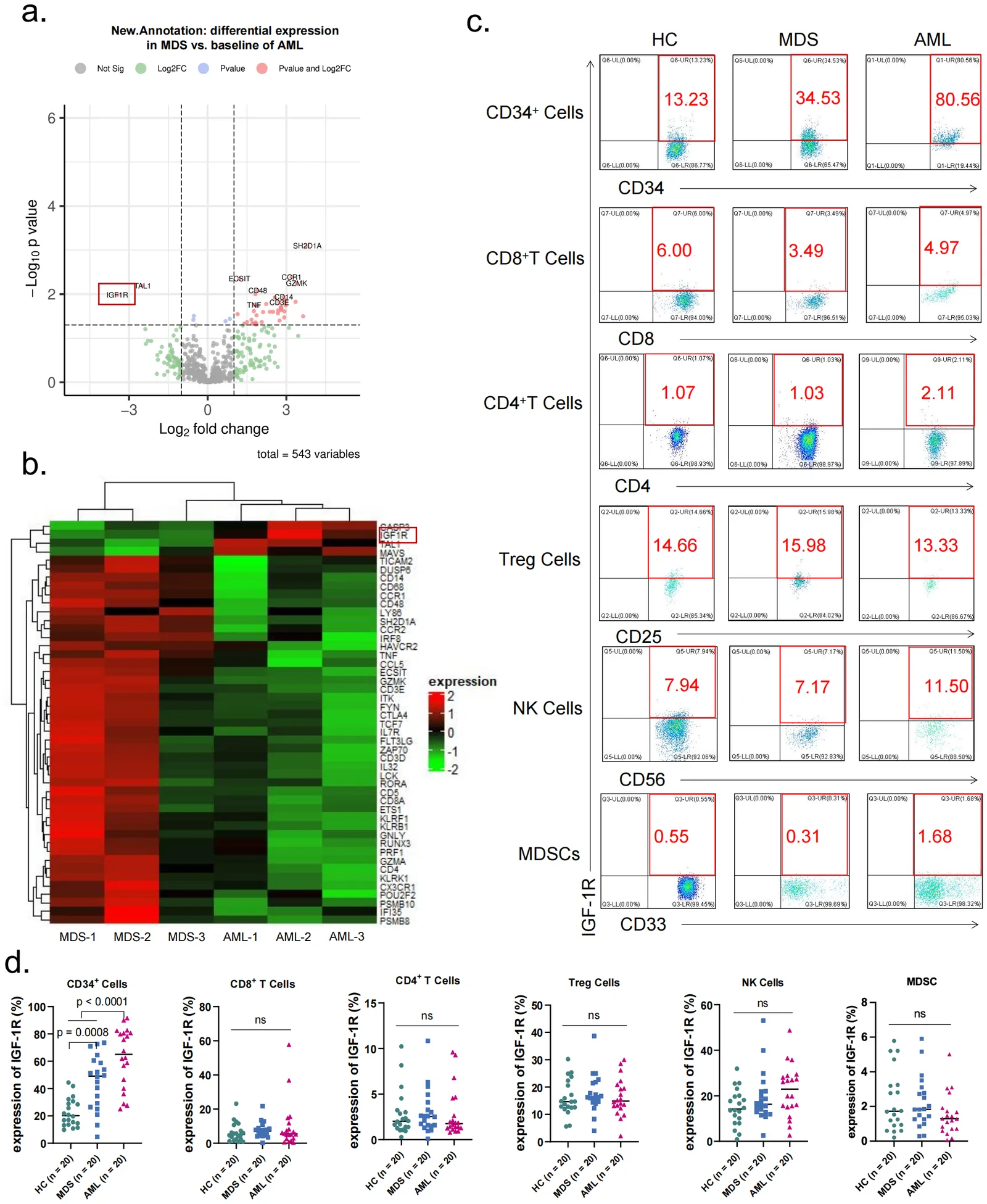
Differential expression of IGF-1R on the surface of CD34+ cells is involved in the onset and development of MDS. **(A, B)** Nanostring sequencing revealed differential expression of IGF-1R in the bone marrow of MDS and AML patients, with significantly higher expression of IGF-1R in the bone marrow of MDS-AML patients than in MDS patients. **(C, D)** HC, MDS patients, and AML patients: expression levels of IGF-1R on the surface of various cells in bone marrow. All data are presented as mean ± SD (n = 20 per group). Statistical significance was assessed using one-way ANOVA followed by Tukey’s *post hoc* test.

### Exogenous IGF-1 promotes the proliferation of MDS cells, and inhibition of IGF-1R can counteract this proliferative effect and induce apoptosis in MDS cells

IGF-1 is the ligand for IGF-1R. Numerous studies have shown that IGF-1 is crucial for maintaining the stemness and self-renewal of various types of stem cells (24, 25). Therefore, we added exogenous IGF-1 to the tumor cell culture system, followed by OSI-906, a highly selective IGF-1R inhibitor, and conducted EdU cell proliferation assays and apoptosis assays. The experimental results showed that exogenous IGF-1 promoted the proliferation of MDS cells, increasing the proliferation rate of SKM-1 cells from (36.13 ± 5.89) % to (51.26 ± 5.48) %, and the proliferation rate of KG-1 cells from (45.34 ± 5.04) % to (60.87 ± 6.00) %. After adding OSI-906, the proportions of proliferating cells decreased to (22.53 ± 4.65) % and (28.37 ± 2.50) %, respectively. This further confirmed the regulatory role of IGF-1/IGF-1R in the proliferation of MDS cells (**Figures 2A–C**). Meanwhile, the results of the apoptosis assay indicated that inhibition of IGF-1R significantly increased the apoptosis rate of MDS cells. The apoptosis rate of SKM-1 cells increased from (8.71 ± 1.12) % to (65.19 ± 7.61) %, and the apoptosis rate of KG-1 cells increased from (3.99 ± 2.66) % to (40.41 ± 6.40) % (**Figures 2D–F**). The results of cell proliferation and apoptosis assays demonstrate that IGF-1/IGF-1R is critical for the survival of MDS cells, and inhibition of IGF-1R effectively suppresses MDS cell proliferation and promotes apoptosis.

**Figure 2:**
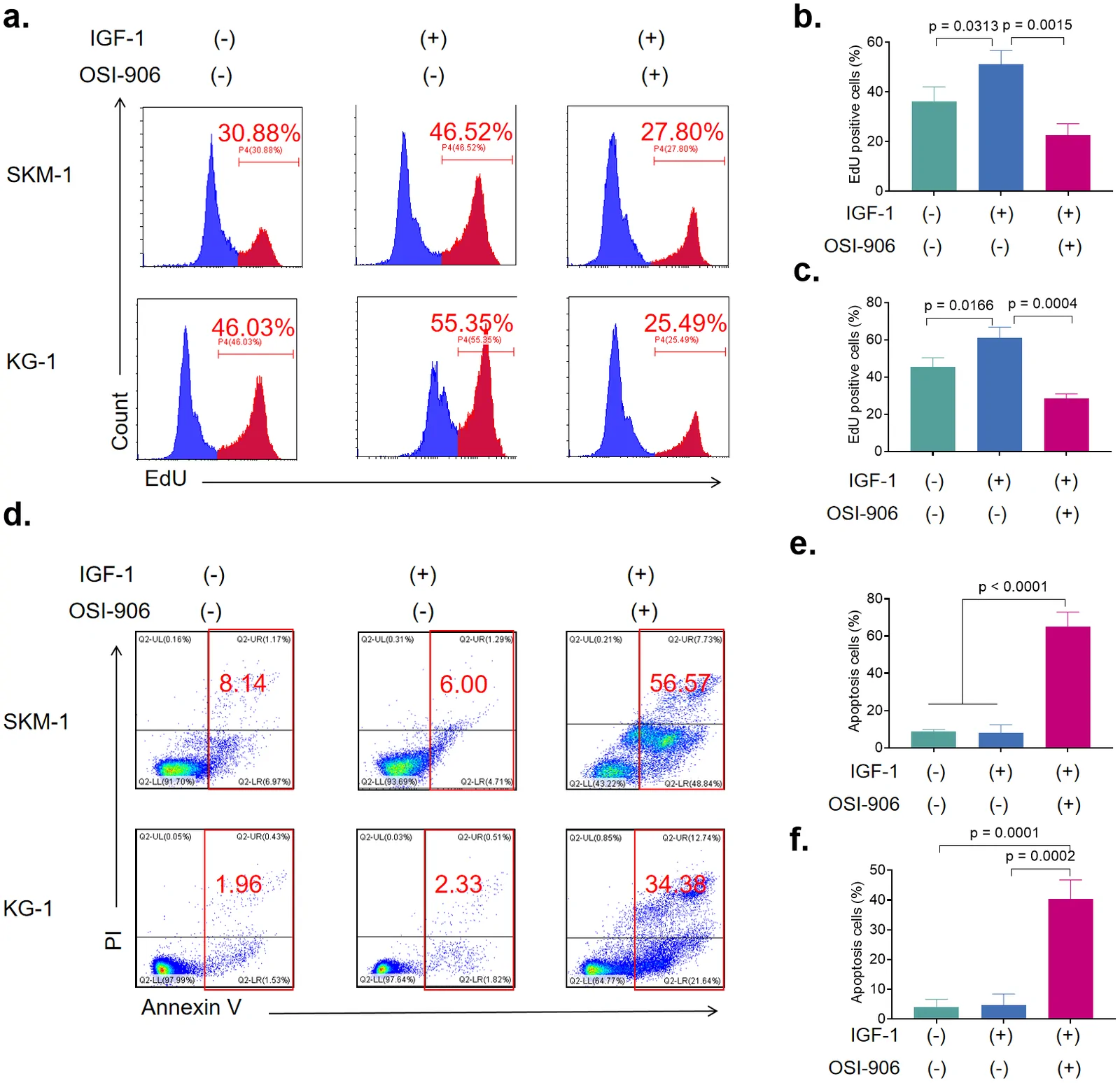
The regulatory role of IGF-1/IGF-1R in the proliferation and apoptosis of MDS cells. **(A)** Exogenous IGF-1 promotes the proliferation of MDS cells, and inhibition of IGF-1R can counteract this effect. Statistical analysis of **(B)** SKM-1 and **(C)** KG-1. **(D)** Inhibition of IGF-1R promotes apoptosis in MDS cells. Statistical analysis of **(E)** SKM-1 and **(F)** KG-1. All data are presented as mean ± SD (n = 3 independent biological replicates). Statistical significance was assessed using one-way ANOVA followed by Tukey’s *post hoc* test.

### Inhibition of IGF-1R induces DNA damage accompanied by an altered DNA damage response and increases the expression of MICA/B in MDS cells

In response to the phenomenon that the proliferation of MDS cells is inhibited and the apoptosis rate increases after inhibiting IGF-1R, we conducted further investigations. We found that inhibition of IGF-1R resulted in DNA damage in MDS cells, as evidenced by increased Tunel staining positivity and elongated comet tails in the comet assay (**Figures 3A–D**). Subsequent Western blot results indicated that inhibition of IGF-1R led to reduced expression of p-PRKDC, while p-ATM and p-ATR expression were upregulated (**Figures 3E, F**). These data suggest that IGF-1R inhibition induces DNA damage that is accompanied by an altered DNA damage response, with increased pATM and pATR and reduced p-PRKDC. Previous studies have shown that after DNA damage occurs in tumor cells, the expression of MICA/B on their surface increases, thereby mediating NK cell-mediated killing (26–28). We simultaneously detected the expression of MICA/B on the surface of MDS cells after IGF-1R inhibition and found that the expression of MICA/B on the surface of treated MDS cells increased. The MICA/B positivity rate on the surface of SKM-1 cells increased from (3.61 ± 1.49) % to (12.41 ± 3.65) %; the MICA/B positivity rate on the surface of KG-1 cells increased from (3.11 ± 1.32) % to (17.64 ± 2.94) % (**Figures 3G, H**). These findings suggest that IGF-1R inhibition induces DNA damage in MDS cells and is accompanied by increased MICA/B expression on the cell surface.

**Figure 3:**
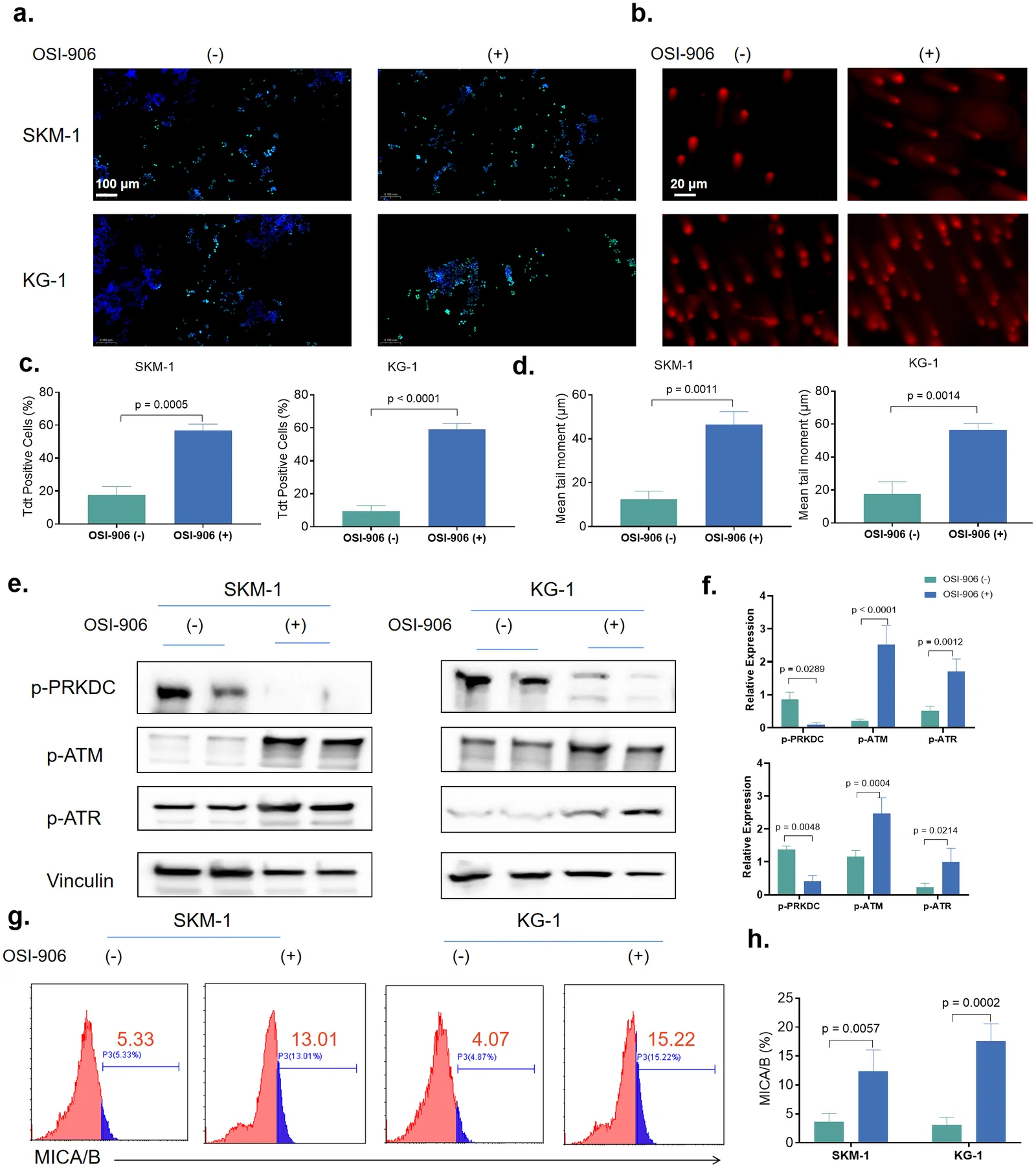
Inhibition of IGF-1R induces DNA damage, alters the expression of DNA damage response markers, and upregulates the expression of MICA/B on the surface of MDS cells. **(A, C)** Tunel assay to detect DNA strand breaks in SKM-1 and KG-1 cells. **(B, D)** Comet assay to detect DNA damage in SKM-1 and KG-1 cells. **(E, F)** Western blot assay to detect DNA damage response proteins (p-ATM, p-ATR, and p-PRKDC) in MDS cells. **(G, H)** Changes in MICA/B expression on the surface of MDS cells detected by flow cytometry after IGF-1R inhibition. All data are presented as mean ± SD (n = 3 independent biological replicates). Statistical significance was assessed using one-way ANOVA followed by Tukey’s *post hoc* test.

### Inhibition of IGF-1R increases NK cell activation markers and increase MDS cell apoptosis in a co-culture system of MDS cells and NK cells

MICA/B is one of the ligands for NKG2D, a receptor that is lowly expressed on normal cells but is upregulated on damaged, transformed, or infected cell surfaces through post-transcriptional or post-translational mechanisms and intracellular pathways. Its binding to NKG2D triggers NK cell effector functions. We discovered that inhibiting IGF-1R induces apoptosis in MDS cells, accompanied by DNA damage and increased MICA/B expression on the cell surface. Could this highly expressed MICA/B contribute to NK cell activation through NKG2D on NK cell surfaces? To explore this conjecture, we established a co-culture system of SKM-1 and NK-92 and treated it with IGF-1R inhibition and then measured the functional indicators of NK-92 and the apoptosis of SKM-1 in the co-culture system (**Figure 4A**; **Supplementary Figure S2**). The results showed that after inhibiting IGF-1R, the NKG2D-positive rate increased from (28.56 ± 5.53)% to (66.98 ± 10.58)% (**Figures 4B, C**), by the mean time, the CD107a-positive rate of NK-92 cells in the co-culture system increased from (27.45 ± 11.27)% to (66.43 ± 13.97)%, and the perforin-positive rate increased from (15.69 ± 6.50)% to (61.93 ± 12.60)% (**Figures 4D, E**), which indicates increased expression of NK cell effector markers. The apoptosis rate of SKM-1 cells in the co-culture system also increased from (24.63 ± 1.85)% to (54.29 ± 7.31)% (**Figures 4F, G**). These findings indicate that IGF-1R inhibition upregulates MICA/B on MDS cells and increases NKG2D expression and effector function in NK cells, supporting the involvement of the MICA/B–NKG2D axis in the enhanced NK cell–mediated killing observed in the co-culture system. This suggests that IGF-1R inhibition may enhance the interaction between MDS cells and NK cells within the bone marrow immune microenvironment of MDS patients, supporting IGF-1R as a potential therapeutic target for MDS.

**Figure 4:**
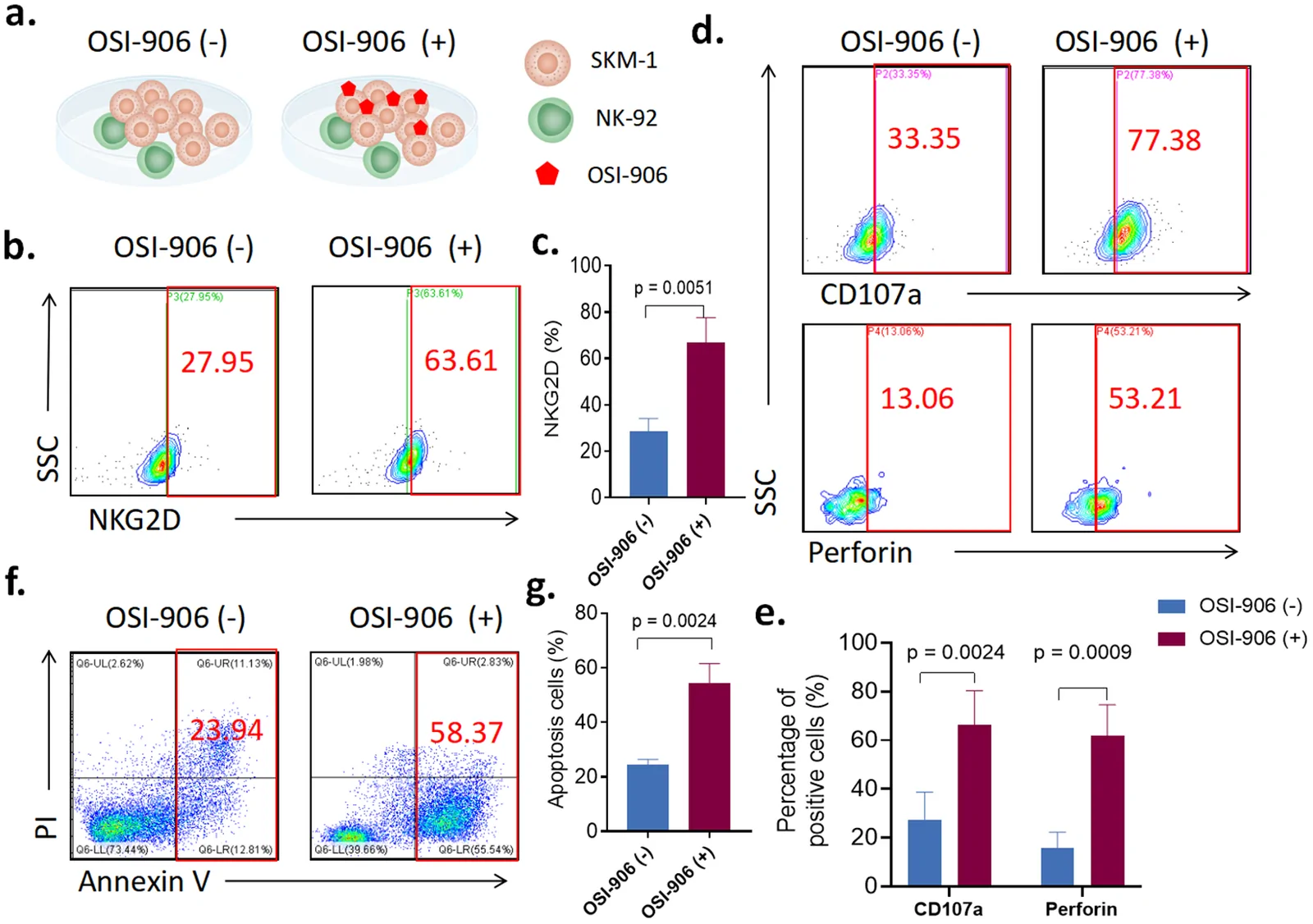
Effects of IGF-1R inhibition in co-culture systems on SKM-1 and NK-92 cells. **(A)** Schematic diagram of co-culture of SKM-1 cells and NK-92 cells. **(B, C)** Inhibition of IGF-1R increases the expression of NKG2D on the surface of NK-92 cells in co-culture systems. **(D, E)** The effect of IGF-1R inhibition in the co-culture system on the expression of the NK cell effector markers CD107a and perforin in NK-92 cells. **(F, G)** The effect of IGF-1R inhibition on the apoptosis rate of SKM-1 cells in a co-culture system. All data are presented as mean ± SD (n = 3 independent biological replicates). Statistical significance was assessed using one-way ANOVA followed by Tukey’s *post hoc* test.

## Discussion

MDS represents a malignant clonal disorder of hematopoietic stem/progenitor cells, with its pathogenesis involving multiple factors including molecular abnormalities, cytogenetic alterations, and immune dysfunction. Among these, the imbalance in the patient’s immune surveillance system and the diminished ability to eliminate abnormal clones lead to immune escape and immune tolerance of malignant clonal cells. This represents one of the key mechanisms driving the progression of MDS to AML. Therefore, studying immune cells within the bone marrow of MDS patients holds significant importance for exploring disease progression mechanisms and identifying potential therapeutic targets. However, immune cells constitute an extremely small proportion of total bone marrow cells, and their numbers further diminish during the immune exhaustion phase of MDS progression. Consequently, therapeutic approaches directly targeting immune cells themselves face limitations. In this study, we investigated whether IGF-1R inhibition could enhance the interaction between MDS cells and NK cells: inhibition of IGF-1R induces DNA damage in MDS cells and is accompanied by increased surface MICA/B expression, a strategy that not only inflicts direct damage on MDS cells but may also increase their susceptibility to NK cell–mediated killing.

In recent years, sequencing cells within bone marrow to identify differentially expressed genes associated with the progression of MDS to AML has proven extremely challenging. Due to the low abundance of target cells—whether MDS cells or various immune cells—in bone marrow, sample processing may also introduce PCR amplification bias. This makes it difficult to detect low-abundance differentially expressed genes or may result in the detection of a large number of statistically insignificant genes. Therefore, in this study, we adopted the Nanostring sequencing method, which is based on the nCounter system that directly counts RNA molecules using fluorescent barcodes, without the need for amplification steps, thereby avoiding PCR-induced bias (29–31). This method is particularly suitable for the precise detection of low-abundance immune-related genes (such as cytokines and chemokines) and provides more reliable analysis of gene expression in various cell subsets (32, 33). Currently, no one has applied this method in MDS research.

Using Nanostring sequencing technology, we identified IGF-1R, a key player in various physiological processes such as cell growth, proliferation, differentiation, and apoptosis. Recent studies have shown that it plays a crucial role in the occurrence, development, and metastasis of cancer. IGF-1R has emerged as a promising and prospective biomarker for cancer diagnosis and treatment. However, its role in MDS has not yet been studied. Through flow cytometry analysis of bone marrow samples from healthy donors, MDS patients, and MDS-AML patients, IGF-1R was found to exhibit significant differences in expression levels on CD34+ cells with disease progression. It should be noted that the NanoString analysis was performed on bulk bone marrow and therefore does not provide cell subset–resolved information; the finding that differential IGF-1R expression is predominantly observed on CD34+ cells is based on the separate flow cytometry analysis in which each bone marrow population was gated and analyzed independently.

As anticipated, the IGF-1/IGF-1R axis plays an important role in MDS cell survival. Inhibiting IGF-1R induces DNA damage in MDS cells, accompanied by an altered DNA damage response, and MICA/B expression on the MDS cell surface is increased in this context. As a potent ligand for NKG2D, MICA/B can activate NK cell function upon binding. *In vitro* co-culture experiments showed increased NKG2D expression on NK-92 cells together with enhanced expression of the effector markers CD107a and perforin, and the apoptosis of SKM-1 cells in co-culture was increased (**Figure 5**). The schematic diagram in **Figure 5** was created with BioGDP.com (34). These observations support the involvement of the MICA/B–NKG2D axis in the enhanced apoptosis of MDS cells in the co-culture system. This strategy may enhance the interaction between malignant cells and NK cells within the bone marrow immune microenvironment, and provides a rationale for targeting IGF-1R as a novel therapeutic approach for MDS.

**Figure 5:**
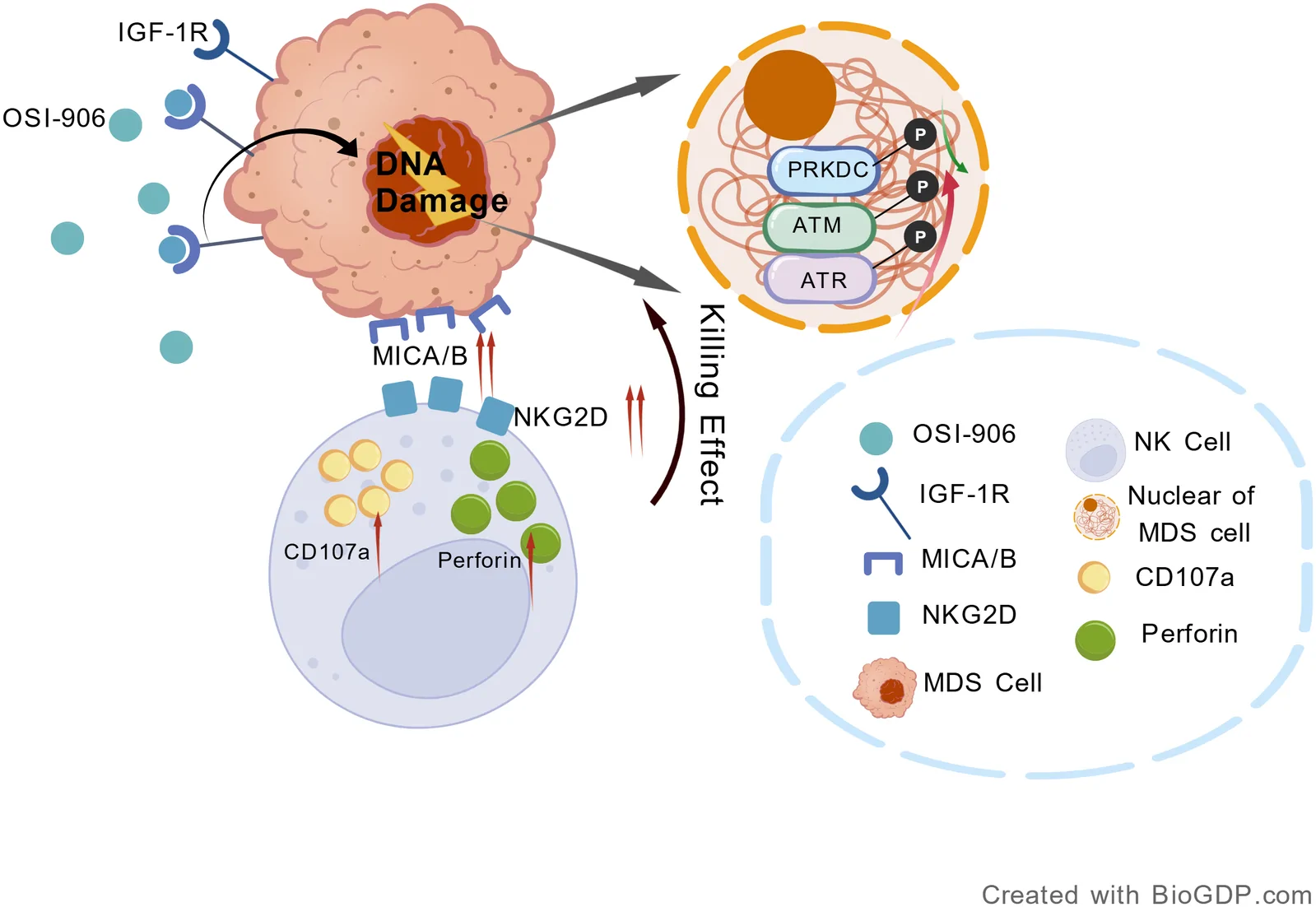
Hypothetical mechanism diagram of the effect of IGF-1/IGF-1R on the interaction between MDS cells and NK cells. Created with BioGDP.com (34).

A question raised during the review process concerns whether IGF-1R upregulation during MDS progression is linked to reduced ferroptosis susceptibility of MDS cells. Notably, megakaryocyte-derived IGF-1 protects hematopoietic stem cells from ferroptosis through mTOR-mediated inhibition of ferritinophagy (35), and ferroptosis has been identified as a critical negative regulator of hematopoietic stem cell maintenance (36). MDS cells have also been reported to be sensitive to ferroptosis induction (37), and IGF-1R-mediated PI3K/AKT signaling has been shown to suppress ferroptosis by enhancing creatine kinase B–GPX4 binding (38). These findings raise the biologically plausible possibility that IGF-1R upregulation could diminish ferroptosis susceptibility of MDS cells and thereby support malignant clonal survival. This hypothesis was not experimentally addressed in the present study and warrants investigation in future work.

This study has several limitations. First, the NanoString analysis was performed on bulk bone marrow in a small discovery cohort and does not provide cell subset-resolved mRNA quantification; the CD34^+^-specific finding is based on independent flow cytometry validation. Second, the mechanistic link between IGF-1R inhibition, DNA damage, MICA/B upregulation, and NKG2D-dependent killing is supported by convergent readouts (TUNEL, comet, Western blot, and co-culture) but not formally proven; blocking or knockdown of MICA/B or NKG2D, assessment of γ-H2AX foci resolution, and functional DNA repair reporter assays will be required. Third, the study is based on MDS cell lines and NK-92 cells, without primary patient samples or *in vivo* models; validation using primary MDS bone marrow cells, primary NK cells, and xenograft or PDX models with human NK cell reconstitution is needed. Finally, IGF-1R upregulation may also affect ferroptosis susceptibility, which was not experimentally addressed here and warrants future investigation.

Together, these findings identify IGF-1R as a potential target for MDS therapy and support the involvement of the MICA/B–NKG2D axis in the interaction between MDS cells and NK cells, pending further validation in primary patient samples and *in vivo* models.

## Data Availability

The raw data supporting the conclusions of this article will be made available by the authors, without undue reservation.

## Supplementary material

Supplementary Figure 1 Gating strategy and IGF-1R expression on bone marrow cell subsets.

Supplementary Figure 2 Schematic of the co-culture experimental design.

Supplementary Table 1 Clinical information of the enrolled patients.

Supplementary Table 2 Antibodies used for flow cytometry.

Supplementary Table 3 Antibodies used for Western blot.
