# Supplementary material for: Insulin-like growth factor-1 receptor participates in the anti-MDS action involving the MICA/B-NKG2D axis
Source: Front Oncol. 2026 Oct 29;16:1960550. doi: 10.3389/fonc.2026.1960550 (PMC13754671; doi:10.3389/fonc.2026.1960550)
Supplement: Supplementary file 1 [file DataSheet1.docx]

**Table S1.** Information about the included patients.

| Sample | Gender | Age | Disease | Type |
| --- | --- | --- | --- | --- |
| HD-1 | Male | 71 | - | - |
| HD-2 | Male | 72 | - | - |
| HD-3 | Male | 61 | - | - |
| HD-4 | Male | 66 | - | - |
| HD-5 | Female | 51 | - | - |
| HD-6 | Female | 66 | - | - |
| HD-7 | Male | 53 | - | - |
| HD-8 | Male | 69 | - | - |
| HD-9 | Female | 69 | - | - |
| HD-10 | Male | 49 | - | - |
| HD-11 | Male | 62 | - | - |
| HD-12 | Male | 78 | - | - |
| HD-13 | Female | 69 | - | - |
| HD-14 | Male | 61 | - | - |
| HD-15 | Female | 76 | - | - |
| HD-16 | Female | 61 | - | - |
| HD-17 | Male | 62 | - | - |
| HD-18 | Female | 72 | - | - |
| HD-19 | Female | 55 | - | - |
| HD-20 | Female | 61 | - | - |
| MDS-1 | Male | 69 | MDS | MDS-5q |
| MDS-2 | Female | 61 | MDS | MDS-5q |
| MDS-3 | Male | 50 | MDS | MDS-5q |
| MDS-4 | Male | 68 | MDS | MDS-IB1 |
| MDS-5 | Male | 74 | MDS | MDS-IB1 |
| MDS-6 | Male | 68 | MDS | MDS-IB1 |
| MDS-7 | Male | 71 | MDS | MDS-IB2 |
| MDS-8 | Female | 52 | MDS | MDS-LB |
| MDS-9 | Male | 61 | MDS | MDS-IB2 |
| MDS-10 | Female | 43 | MDS | MDS-IB2 |
| MDS-11 | Female | 77 | MDS | MDS-IB1 |
| MDS-12 | Male | 53 | MDS | MDS-LB |
| MDS-13 | Male | 75 | MDS | MDS-IB2 |
| MDS-14 | Female | 60 | MDS | MDS-IB2 |
| MDS-15 | Male | 61 | MDS | MDS-IB2 |
| MDS-16 | Female | 68 | MDS | MDS-IB2 |
| MDS-17 | Female | 65 | MDS | MDS-IB1 |
| MDS-18 | Female | 79 | MDS | MDS-5q |
| MDS-19 | Female | 73 | MDS | MDS-IB2 |
| MDS-20 | Male | 65 | MDS | MDS-IB2 |
| AML-1 | Male | 72 | AML | M2b |
| AML-2 | Female | 63 | AML | M3 |
| AML-3 | Male | 79 | AML | M3 |
| AML-4 | Male | 61 | AML | M3 |
| AML-5 | Female | 62 | AML | M3 |
| AML-6 | Male | 49 | AML | M4 |
| AML-7 | Male | 71 | AML | M4 |
| AML-8 | Female | 63 | AML | M3 |
| AML-9 | Male | 68 | AML | M4 |
| AML-10 | Male | 63 | AML | M4 |
| AML-11 | Male | 74 | AML | M5 |
| AML-12 | Male | 72 | AML | M3 |
| AML-13 | Female | 56 | AML | M5 |
| AML-14 | Female | 54 | AML | M2b |
| AML-15 | Male | 79 | AML | M4 |
| AML-16 | Male | 59 | AML | M3 |
| AML-17 | Female | 71 | AML | M4 |
| AML-18 | Female | 58 | AML | M4 |
| AML-19 | Female | 54 | AML | M4 |
| AML-20 | Male | 63 | AML | M3 |

**Table S2.** Antibodies for flow cytometry.

| REAGENT | SOURCE | IDENTIFIER |
| --- | --- | --- |
| MICA/B-FITC | Abclonal | A25276 |
| BD Pharmingen^TM^ FITC Annexin V Apoptosis Detection Kit I | BD Pharmingen | AB_2869082 |
| PerCP anti-human CD34 Antibody | Biolegend | Cat#343520 |
| [FITC anti-human CD8 Antibody](https://www.biolegend.com/en-gb/products/fitc-anti-human-cd8-antibody-6149) | Biolegend | Cat#344704 |
| [APC anti-human CD33 Antibody](https://www.biolegend.com/en-us/products/pe-anti-human-cd107a-lamp-1-antibody-4967) | Biolegend | Cat#381604 |
| [APC/Cyanine anti-human CD56 (NCAM) Antibody](https://www.biolegend.com/en-us/products/pe-anti-human-cd107a-lamp-1-antibody-4967) | Biolegend | Cat#362512 |
| [Percp anti-human CD4 Antibody](https://www.biolegend.com/en-us/products/pe-anti-human-cd107a-lamp-1-antibody-4967) | Biolegend | Cat#300528 |
| [APC anti-human CD25 Antibody](https://www.biolegend.com/en-us/products/pe-anti-human-cd107a-lamp-1-antibody-4967) | Biolegend | Cat#302610 |
| PE anti-human CD221 (IGF-1R) Antibody | Biolegend | Cat#351806 |
| [PE anti-human CD107a (LAMP-1) Antibody](https://www.biolegend.com/en-us/products/pe-anti-human-cd107a-lamp-1-antibody-4967) | Biolegend | Cat#328608 |
| [Percp/Cynine5.5 anti-human Perforin Antibody](https://www.biolegend.com/en-us/products/fitc-anti-human-perforin-antibody-884) | Biolegend | Cat#308114 |
| [Brilliant Violet 421TM anti-human/mouse Granzyme B Recombinant Antibody](https://www.biolegend.com/en-gb/products/brilliant-violet-421-anti-humanmouse-granzyme-b-recombinant-antibody-17577) | Biolegend | Cat#396414 |
| Brilliant Violet 421™ anti-human FOXP3 Antibody | Biolegend | Cat#320124 |
| [APC anti-human CD3 Antibody](https://www.biolegend.com/en-us/products/pe-anti-human-cd107a-lamp-1-antibody-4967) | Biolegend | Cat#317318 |
| PerCP anti-mouse/human CD11b Antibody | Biolegend | Cat#101230 |
| [FITC anti-human HLA-DR Antibody](https://www.biolegend.com/en-us/products/fitc-anti-human-perforin-antibody-884) | Biolegend | Cat#327006 |
| APC anti-human CD45 Antibody | Biolegend | Cat#304012 |

**Table S3.** Antibodies for Westernblot.

| REAGENT | SOURCE | IDENTIFIER |
| --- | --- | --- |
| Phospho-DNA-PKcs (Ser2056) | CST | #68716T |
| Phospho-ATR (Thr1989) | CST | #30632T |
| Phospho-ATM (Ser1981) | CST | #5883T |
| Vinculin | CST | #4650T |
| Anti-rabbit IgG, HRP-linked | CST | #7047S |
| Anti-mouse IgG, HRP-linked | CST | #7076S |

Figure S1. Flow labeling strategies for various cell types (a) CD34+ cells

, (b) Treg cells, (c) CD8+T cells, (d) CD4+T cells, (e) NK cells, (f) MDSCs in bone marrow

Figure S2. Flow cytometry labeling in a co-culture system of SKM-1 and NK-92 cells.
